# Metagenomic Analysis of Fever, Thrombocytopenia and Leukopenia Syndrome (FTLS) in Henan Province, China: Discovery of a New Bunyavirus

**DOI:** 10.1371/journal.ppat.1002369

**Published:** 2011-11-17

**Authors:** Bianli Xu, Licheng Liu, Xueyong Huang, Hong Ma, Yuan Zhang, Yanhua Du, Pengzhi Wang, Xiaoyan Tang, Haifeng Wang, Kai Kang, Shiqiang Zhang, Guohua Zhao, Weili Wu, Yinhui Yang, Haomin Chen, Feng Mu, Weijun Chen

**Affiliations:** 1 Center for Disease Control and Prevention of Henan Province, Zhengzhou, China; 2 Beijing Genomics Institute in Wuhan, Wuhan, China; 3 Key Laboratory of Genome Sciences and Information, Beijing Institute of Genomics, Chinese Academy of Sciences, Beijing, China; 4 Center for Disease Control and Prevention of Xinyang City, Xinyang, China; 5 State Key Laboratory of Pathogen and Biosecurity, Institute of Microbiology and Epidemiology, Academy of Military Medical Sciences, Beijing, China; George Mason University, United States of America

## Abstract

Since 2007, many cases of fever, thrombocytopenia and leukopenia syndrome (FTLS) have emerged in Henan Province, China. Patient reports of tick bites suggested that infection could contribute to FTLS. Many tick-transmitted microbial pathogens were tested for by PCR/RT-PCR and/or indirect immunofluorescence assay (IFA). However, only 8% (24/285) of samples collected from 2007 to 2010 tested positive for human granulocytic anaplasmosis (HGA), suggesting that other pathogens could be involved. Here, we used an unbiased metagenomic approach to screen and survey for microbes possibly associated with FTLS. BLASTx analysis of deduced protein sequences revealed that a novel bunyavirus (36% identity to Tehran virus, accession: HQ412604) was present only in sera from FTLS patients. A phylogenetic analysis further showed that, although closely related to Uukuniemi virus of the *Phlebovirus* genus, this virus was distinct. The candidate virus was examined for association with FTLS among samples collected from Henan province during 2007–2010. RT-PCR, viral cultures, and a seroepidemiologic survey were undertaken. RT-PCR results showed that 223 of 285 (78.24%) acute-phase serum samples contained viral RNA. Of 95 patients for whom paired acute and convalescent sera were available, 73 had serologic evidence of infection, with 52 seroconversions and 21 exhibiting a 4-fold increase in antibody titer to the virus. The new virus was isolated from patient acute-phase serum samples and named Henan Fever Virus (HNF virus). Whole-genome sequencing confirmed that the virus was a novel bunyavirus with genetic similarity to known bunyaviruses, and was most closely related to the Uukuniemi virus (34%, 24%, and 29% of maximum identity, respectively, for segment L, M, S at maximum query coverage). After the release of the GenBank sequences of SFTSV, we found that they were nearly identical (>99% identity). These results show that the novel bunyavirus (HNF virus) is strongly correlated with FTLS.

## Author Summary

Initially in 2007, and again between 2008 and 2010, cases of a life-threatening disease with sudden fever, thrombocytopenia, and leukopenia were reported in Henan Province, China. Patient reports of tick bites suggested that this disease could be infectious or tick-transmitted. Many patients were provisionally diagnosed with human granulocytic anaplasmosis (HGA). However, only 24 of 285 (8%) had objective evidence of HGA, suggesting that other pathogens likely contributed to fever, thrombocytopenia and leukopenia syndrome (FTLS). Illumina sequencing was used for direct detection in clinical samples of pathogens possibly associated with FTLS. A novel bunyavirus was found only in samples from FTLS patients. Further epidemiologic and laboratory investigation confirmed that the novel bunyavirus was associated with FTLS. The results illustrate that metagenomic analysis is a powerful method for the discovery of novel pathogenic agents. Combined with epidemiologic investigation, it could assist in rapid diagnosis of unknown diseases and distinguish them from other diseases with similar symptoms caused by known pathogens.

## Introduction

In May 2007, a county hospital in Xinyang City, Henan Province treated three patients with fever, abdominal pain, bloating, nausea, vomiting, gastrointestinal bleeding, and elevated aminotransferases. The local hospital diagnosed the disease as acute gastroenteritis. A family member of one patient reported the disease to the Henan Center for Disease Control and Prevention (CDC), which sent a team to investigate. The investigation revealed that the disease had the following characteristics: (1) acute onset with fever; (2) low white blood cell and platelet counts; (3) high levels of alanine and aspartate transaminases; (4) positive urine protein. On the basis of these features, the Henan CDC excluded the possibility of gastrointestinal disorders. In order to identify the disease etiology, the Henan CDC team used the above clinical characteristics as the case definition to search for similar cases in local hospitals in this and neighboring counties, while establishing a disease surveillance system that required all medical institutions to report cases that met the above case definition. Altogether, 79 cases were found in 2007 in Henan, with 10 fatalities (case fatality rate, 12.7%). All patients were farmers and resided in mountainous or hilly villages, and many had reported tick bites 7–9 days before illness, further suggesting an infectious etiology. In recent years, patients with similar clinical symptoms were reported with human granulocytic anaplasmosis (HGA; *Anaplasma phagocytophilum*) in neighboring Anhui province [1]. In 2005, there was an epidemic of Tsutsugamushi (scrub typhus/*Orientia tsutsugamushi*) in this area [2]. Clinical investigations, epidemiological analyses, and laboratory testing prompted consideration of rickettsial diseases as possible causes, including HGA, human monocytic ehrlichiosis (HME; *Ehrlichia chaffeensis*), and Tsutsugamushi disease. Specific methods such as polymerase chain reaction (PCR) and immunofluorescence assays (IFAs) for these pathogens were then used to determine if these cases were attributable to HGA or HME [3], [4]. However, only 18 of 79 (22.7%) patients were positive for *A. phagocytophilum* based on serology and DNA testing. Thus, the disease was initially considered at least partly caused by *A. phagocytophilum*, and cases were provisionally diagnosed as suspected HGA based on clinical and epidemiological data [1]–[3], [5]–[7]. In the 3 years since 2007, 206 suspected cases have been discovered in Henan, but there was only a very low positive rate of *A. phagocytophilum* confirmation (6 of 206 patients) and no pathogen was isolated. Similar cases were also reported in the mountainous and hilly areas of nearby Shandong, Jiangsu, Hubei and Anhui provinces, indicating that the disease already existed for some time and was widely distributed [7]. We decided to address the possible causative pathogen underlying this infection.

On the basis of epidemiological and clinical characteristics, we considered two types of diseases to be possible: rickettsial and arthropod-borne viral disease. Because of the low rates of *A. phagocytophilum* (rickettsial disease) detection, the research team intensified its virus search to take into account arthropod-borne viruses, including *Flaviviridae* (Dengue viruses [DENV], Japanese encephalitis virus [JEV]), *Togaviridae* (Chikungunya virus, Eastern equine encephalitis virus [EEEV], Western equine encephalitis virus), and *Bunyaviridae* (Crimean-Congo hemorrhagic fever virus, Hantaan virus, and Rift valley fever virus) [8]. Specific PCR assays for these viruses were used [9]–[16]. However, none of the patients from 2007 to 2010 was positive for these viruses, suggesting a new infectious agent, possibly a virus, remained to be discovered. Thus, the syndrome was considered an emerging infectious disease and was named fever, thrombocytopenia and leukopenia syndrome (FTLS). To identify the etiology, the research team adopted the following strategy: 1) sequencing of randomly amplified cDNA/DNA from FTLS patient samples using high-throughput Illumina sequencing to specifically explore viral communities present in patients suffering from FTLS, 2) PCR detection of target DNA directly from clinical specimens, 3) viral culture, 4) immunodetection methods, and 5) electron microscopic study of the morphology of the cultured virus.

Culture followed by serological and molecular tests is a standard approach for identifying an unknown virus. However, culture of an unknown virus is time-consuming, even taking several years to confirm a novel infection like HIV [17]. Otherwise, virus culture often fails because of the lack of cell lines capable of supporting propagation of viruses (e.g., hepatitis B and C virus). Methods for cloning nucleic acids of microbial pathogens directly from clinical samples offer opportunities for pathogen discovery, thereby laying the foundation for future studies aimed at assessing whether novel or unexpected viruses play a role in disease etiology. Random PCR and subtractive cloning sequencing have identified previously unknown pathogens as etiological agents of several acute and chronic infectious diseases [18], [19]. Recently, high-throughput sequencing approaches have been used for pathogen detection and discovery in clinical samples [20]–[22]. We also developed a method for exploring viruses, both known and novel, using high-throughput Illumina sequencing. In this study, high-throughput Illumina sequencing was applied to specifically explore the viral communities in patients with FTLS, using healthy subjects as controls. Here, we provide evidence for the discovery of a novel bunyavirus associated with FTLS through high-throughput sequencing. Subsequent culture of the virus and PCR detection of the specific virus in patient specimens confirmed these findings.

## Materials and Methods

### Ethics statement

This research was approved by the Review Board of the Center for Disease Control and Prevention of Henan Province, the Review Board of the Center for Disease Control and Prevention of Xinyang city, the Review Board of Beijing Institute of Genomics, the Review Board of Beijing Genomics Institute in Shenzhen, and the Review Board of the Institute of Microbiology and Epidemiology. All participants gave written informed consent for use of their samples in research.

### Biosafety

Given the serious nature of FTLS, it was decided to handle all clinical specimens and perform all experiments involving live virus in a biosafety level-3 (BSL-3) facility.

### Clinical samples

We studied 285 Henan province patients with FTLS whose samples were submitted to the Henan Province CDC between May 2007 and July 2010. Acute-phase serum samples from all patients were collected. Paired convalescent sera were available from 95 patients.

### Diagnostic testing of sera for microbial agents possibly related to FTLS

Sera were tested by reverse transcription (RT)-PCR, PCR, and/or indirect IFA serological assays for a number of microbial agents, including *A. phagocytophilum*, *Ehrlichia chaffeensis*, Dengue fever virus, Japanese encephalitis virus, Chikungunya virus, Eastern equine encephalitis virus, Western equine encephalitis virus, Crimean-Congo hemorrhagic fever virus, Rift Valley fever virus, Sandfly fever Naples Sabin virus, and Hantavirus [3]–[6], [9]–[16], [23]. Antigen slides for diagnosis of HGA (*A. phagocytophilum*) were purchased from Focus Diagnostics (IF1450G, CA, USA). Antigen slides for diagnosis of other pathogens were prepared by our laboratories. Fluorescein isothiocyanate (FITC)-conjugated goat anti-human IgG (Fc) was purchased from Sihuan Sci-Technics Company (Beijing, China).

### Sample processing for Illumina sequencing

Equal quantities (100 µL) of acute-phase sera from 10 FTLS patients who had a history of tick bite were pooled and centrifuged at 1000 x g for 10 minutes. The supernatant was collected for DNA and RNA extraction. The same was done for 10 sera from healthy subjects (control).

### DNA and RNA extraction

DNA was extracted from 140 µL of each sample using the QIAamp DNA mini Kit (Qiagen, 51304) according to the manufacturer's instructions. DNA was eluted from the columns with 50 µL water containing 20 µg/mL RNaseA. After incubation at 37°C for 15 minutes to eliminate RNA, DNA was used immediately or stored at −80°C.

Total RNA was extracted from 140 µL of each sample using the QIAamp viral RNA mini Kit (Qiagen, 52904) according to the manufacturer's instructions. RNA was eluted from the columns with 50 µL of diethyl pyrocarbonate (DEPC)-treated water containing 1 U DNaseI. Samples were incubated at 37°C for 15 minutes to eliminate human DNA, followed by DNase inactivation at 95°C for 10 minutes. RNA was used immediately or stored at −80°C.

### Reverse transcription

cDNA was synthesized from 6 µL of RNA by reverse transcription at 45°C for 50 minutes in a 20-µL solution containing 50 mM Tris–HCl (pH 8.3), 75 mM KCl, 3 mM MgCl_2_, 10 mM DTT, 100 ng of random hexamer primers, 200 U of Superscript II (Invitrogen, 18064–014), 25 U of RNasin (Promega, N2511), and 0.5 mM dNTPs.

### Random hexamer PCR

Random hexamer PCR was carried out in a 25-µL mixture containing 4 µL of cDNA or DNA, 10 mM Tris-HCl (pH 8.4), 50 mM KCl, 2.5 mM MgCl_2_, 100 µM dNTPs, 1 U Taq DNA Polymerase (Promega, M1661) and 100 ng of random hexamer primers containing a linker (5'-GCCGGAGCTCTGCAGAATTCNNNNNN-3'). After denaturing at 95°C for 5 minutes, targets were amplified by 45 cycles of 95°C for 30 seconds, 40°C for 30 seconds, 50°C for 30 seconds, and 72°C for 90 seconds. The amplified products were detected by agarose gel electrophoresis. Pure water was used as a negative control.

### High-throughput sequencing by the Illumina method

FTLS patient and control genomic DNA/cDNA libraries were constructed according to the manufacturer's instructions (Illumina). In brief, RT-PCR and PCR products were roughly quantified by UV absorption and equal amounts of each sample were mixed. Nucleic acids within the mixtures were sheared by sonication, and fragments in the 150–180-bp range were collected by cutting bands from an agarose gel after electrophoresis. The sheared DNA and cDNA ends were repaired using Klenow DNA polymerase, after which 5' termini were phosphorylated and 3' termini were polyadenylated. The adaptors were added, PCR enrichment was performed, and 150–180-bp fragments were collected for sequencing by the Illumina method.

The sequencing procedure was performed according to the manufacturer's instructions (Illumina). In this process, template library DNA was hybridized to the surface of the flow cells and multiple copies of DNA were made to form clusters using the Illumina cluster station. Workflow steps included template hybridization, isothermal amplification, linearization, and final denaturation and hybridization of sequencing primers. Paired-end sequencing (100 cycles) was performed using a four-color DNA Sequencing-By-Synthesis (SBS) technology following the manufacturer's instructions.

Short Oligonucleotide Analysis Package (SOAP) was used to handle the large amounts of short reads generated by parallel sequencing [24]. Briefly, after filtering out highly repetitive sequences and adaptor sequences, the overlapping datasets between FTLS and healthy subjects were analyzed by subtracting fragments that mapped to both host genomic-plus-transcript and bacteria databases. The non-redundant reads were mapped onto a virus database downloaded from NCBI (ftp://ftp.ncbi.nih.gov/genbank/). The resulting alignments were filtered to identify unique sequences by examining alignment (identity ≥80%) and E-value scores (e≤10^−2^). Filtered unique alignments were examined in the taxonomy database (NCBI) using a custom software application written in Perl (BioPerl version 5.8.5). Unmapped reads were examined in GenBank nucleic acid and protein databases using BLASTn and BLASTx, respectively [25]–[27]. Unique alignments were examined in the taxonomy database (NCBI). Sequences without hits were placed in the ‘‘unassigned’’ category. Sequences were phylotyped as human, bacterial, phage, viral, or other based on the identity of the best BLAST hit. Considering misannotation and low-complexity for Illumina short reads, sequences assigned to the same virus family were further assembled into contigs with Velvet 1.1.04 (K-mer length  = 21; coverage cutoff: default 0; Insert length: PE only; minor contig length: 42) for direct comparison with GenBank nucleic acid databases using BLASTn [28]. Contigs were also blasted with GenBank protein databases using BLASTx [28]. An E-value cutoff of 1×10^−5^ was applied to both BLASTn and BLASTx analyses. Sequences phylotyped as viral were placed in the “viral” category.

### RT-PCR detection of the bocavirus

Our mass sequencing data revealed that some sequences showed possible infection with human bocavirus, which belongs to the *Parvoviridae* family. PCR performed to detect human bocavirus tentatively identified in sera from FTLS patient samples amplified a 291-bp fragment of the NS1 gene, as described previously [29]. Amplified products were detected by agarose gel electrophoresis and sequenced using an ABI 3730 DNA Sequencer.

### RT-PCR detection of the novel bunyavirus

Our mass sequencing data revealed a 168-bp sequence (C361, Accession: HQ412604) indicating the possible presence of a novel virus with closest identity (i.e., lowest E-value) to *Tehran virus* which belongs to the *Phlebovirus* genus of the *Bunyaviridae* family. We developed a PCR strategy based on the 168-bp sequence identified in sera from FTLS patient samples to detect the novel bunyavirus using forward (PF: 5'-GAC ACG CTC CTC AAG GCT CT-3') and reverse (PR: 5'-GCC CAG TAG CCC TGA GTT TC-3') primers designed with Primer3 (Supplementary Figure S1). PCR was carried out in a 25-µL mixture containing 4 µL of cDNA, 10 mM Tris–HCl (pH 8.4), 50 mM KCl, 2.5 mM MgCl_2_, 100 µM dNTPs, 1 U Taq Pol (Promega, M1661), 0.25 µM forward primer, and 0.25 µM reverse primer. Thermocycling conditions were as follows: 95°C for 4 minutes (denaturation), followed by 35 cycles of 94°C for 30 seconds, 54°C for 30 seconds, and 72°C for 30 seconds. Amplified products were detected by agarose gel electrophoresis and sequenced using an ABI 3730 DNA Sequencer.

### Virus isolation

The Vero E6 cell line (African green monkey kidney cell) was selected for isolation of the novel bunyavirus associated with FTLS because it supports the growth of many bunyaviruses [30], [31]. Vero E6 cell lines were inoculated with six serum samples that contained novel bunyavirus RNA. Each sample underwent at least three cell culture passages in Vero E6 cell line before being considered negative. Medium was replenished on day 7, and cultures were terminated 14 days after inoculation. All cultures were observed daily for cytopathic effect (CPE). Virus-infected cells and uninfected cells were also examined for the novel bunyavirus by RT-PCR at each passage. Vero cell cultures with obvious CPE and containing novel bunyavirus RNA were further analyzed by morphology, genome sequencing, and serology.

### Transmission electron microscopy

Cells showing CPE and containing novel bunyavirus RNA were collected for thin-section electron microscopy. After discarding the culture supernatant, virus-infected cells (50 mL) were mixed 1∶1 with 4% glutaraldehyde (paraformaldehyde), placed onto Formvar-carbon-coated grids, and stained with 1% methylamine tungstate. Specimens for thin-section electron microscopy were prepared by dehydrating washed cell pellets with serial dilutions of acetone and embedding in epoxy resin. Ultrathin sections were cut on an Ultracut LKBV ultramicrotome, stained with uranyl acetate and lead citrate, and examined under a transmission electron microscope (JEM-1400).

### Genome sequencing

The medium from 20 mL of novel bunyavirus-infected Vero E6 cells was centrifuged at 1,000 x g for 10 minutes and then at 4,000 x g for 10 minutes, after which the supernatant was collected. PEG8000 was added to the supernatant at a final concentration of 10% (w/v) followed by centrifugation at 20,000 x g for 2 hours. The pellet was resuspended in 2 mL 1× phosphate-buffered saline (PBS) for RNA extraction. Random RT-PCR was performed, and the products (500–1500 bps) were collected and ligated into the pGEM-T vector (Promega, A3600) by incubating overnight at 16°C. *Escherichia coli* JM109 were transformed with the ligation mixture and cultured on LB agar containing X-gal. White clones were sequenced using an ABI 3730 DNA Sequencer. Contaminating human and extraneous sequences were eliminated using CrossMatch, and the complete sequence was assembled using Phred-Phrap-Consed [32]. Bridge RT-PCR was employed for gap-closure.

### Phylogenetic analysis

Phylogenetic analyses were performed using the neighbor-joining method in the MEGA software package, version 4.0.2 [33]. Available nucleotide or protein sequences from known viruses were obtained from GenBank for inclusion in the phylogenetic trees. Selected sequences from GenBank included those with the greatest similarity to the sequence read in question based on BLAST alignments as well as representative sequences from all major taxa within the relevant *Bunyaviridae* family. To further establish the relationships between the new virus and the members of the *Phleboviruses* genus, we included all sequences for phleboviruses available in GenBank. Branching orders of the phylograms were verified statistically by resampling the data 1,000 times in a bootstrap analysis with the branch-and-bound algorithm, as applied in MEGA.

### Serologic detection by indirect IFA

After successful isolation of the novel bunyavirus, we developed an indirect IFA to detect specific antibodies in patient serum specimens, as previously described [4]. In brief, monolayers of virus-infected Vero E6 cells showing CPE and containing novel bunyavirus RNA were harvested, and one volume of infected cells was mixed with 0.5 volumes of non-infected cells. The mixture was centrifuged at 1,000 x g for 10 minutes, after which cells were resuspended in 1× PBS, spotted onto 12-well glass slides, and fixed with acetone for 10 minutes. Sera from patients with FTLS (including 285 acute-phase samples and 95 paired sera), patients with respiratory diseases (80 serum samples), and healthy subjects (50 serum samples) were applied to the cells. Samples (diluted 1∶20 in PBS) were screened by first spotting 50 µL of each serum sample per well and incubating for 30 minutes at 37°C. After washing for 10 minutes in PBS, 20 µL of FITC-conjugated goat anti-human IgG (Sihuan Sci-Technics Company, Beijing, China) diluted 1∶40 in buffer containing Evans blue was added to each well and incubated for 30 minutes. After washing, slides were mounted in glycerin and examined by immunofluorescence microscopy. A titer of 1∶20 was considered positive.

## Results

### Epidemiology and clinical features of the disease

All 285 patients with FTLS were from the Henan Province of China and were provisionally diagnosed as suspected HGA on the basis of similar clinical manifestations [5], [7]. They represented four different epidemiologically linked sporadic cases and a few clusters of cases including 79 patients in 2007, seven patients in 2008, 47 patients in 2009, and 152 patients in 2010. The patients presented mainly between April and October, peaking in April-May during the tea-picking season in Henan. All patients resided in mountainous and hilly rural areas. In our study, 238 of 285 patients tested positive for novel bunyavirus infection by RT-PCR and/or IFA.

### Epidemiology and clinical features of the disease in laboratory-confirmed patients

The median age of patients was 57.2 years (range, 23–88) and the male-to-female ratio was 1 to 2.27; 219 patients (92.02%) were farmers and 19 (7.98%) were workers or students. Among patients, 52 (21.85%) reported a tick bite within 2 weeks (5–14 days) before the onset of clinical manifestations; the remaining patients did not recall receiving a tick bite.

The main clinical features in confirmed patients included sudden onset of fever (>37.5°C −40°C) lasting up to 10 days, fatigue, anorexia, headache, myalgia, arthralgia, dizziness, enlarged lymph nodes, muscle aches, vomiting and diarrhea, upper abdominal pain, and relative bradycardia (Table 1). A small number of cases suffered more severe complications, including hypotension, mental status alterations, ecchymosis, gastrointestinal hemorrhage, pulmonary hemorrhage, respiratory failure, disseminated intravascular coagulation, multiple organ failure, and/or death. Most patients had a good outcome, but elderly patients and those with underlying diseases, neurological manifestations, coagulopathy, or hyponatremia tended to have a poorer outcome.

**Table 1:** Symptoms of 238 patients with FTLS at presentation.

Clinical symptoms	Number (%)
Fever	232 (97.48)
Fatigue	223 (93.70)
Anorexia	219 (92.02)
Body sores	193 (81.09)
Nausea	181 (76.05)
Myalgia	165 (69.33)
Coarse breathing sounds	170 (71.43)
Chill	171 (71.85)
Diarrhea	156 (65.55)
Dizziness	164 (68.91)
Vomiting	153 (64.29)
Headache	146 (61.34)
Abdominal pain	134 (56.30)
Enlargement of lymph nodes	128(53.78)
Cough	119 (50.00)
Sputum production	89 (37.39)
Arthralgia	80 (33.61)
Skin rash	19 (7.98)

Laboratory tests showed that confirmed patients characteristically developed thrombocytopenia, leukopenia, proteinuria, and elevated serum aspartate aminotransferase (AST) and alanine aminotransferase (ALT) levels (Table 2). Biochemical tests revealed generally higher levels of lactate dehydrogenase, creatine kinase, AST and ALT enzymes, especially AST.

**Table 2:** Initial laboratory findings of 238 patients with FTLS.

Laboratory variables	Mean (range)	Normal range	Number of abnormal (%)
White-cell count	2.31(2.19–2.42)	4–11×10^9^/L	
Leukopenia			234 (98.32)
Platelet count	58.89(55.26–62.51)	100–300×10^9^/L	
Thrombocytopenia			232 (97.48)
Alanine aminotransferase (ALT)	307.50(256.42–358.58)	0–40 U/L	
Elevated alanine aminotransferase			234 (98.32)
Aspartate aminotransferase (AST)	349.14(294.20–404.08)	0–40 U/L	
Elevated aspartate aminotransferase			234 (98.32)
Lactate dehydrogenase(LDH)	532.95(482.72–583.17)	135–225 U/L	
Elevated lactate dehydrogenase			221 (92.86)
Positive urine protein			205 (86.13)
Calcium	1.94(1.89–1.99)	2.3–2.7 mmol/L	
Low calcium			206 (86.55)
Creatine kinase (CK)	53.24(45.82–60.67)	0–25 U/L	
Elevated creatine kinase			166 (69.75)
Creatine phosphokinase (CPK)	481.66(387.68–575.65)	24–195 U/L	
Elevated creatine phosphokinase			149 (62.61)
Sodium	133.71(132.29–135.13)	135–145 mol/L	
Low sodium			113 (47.48)

### Metagenomic analysis and identification of unknown pathogen

One lane for each of the two sample pools (FTLS and healthy controls), each consisting of 10 samples, was sequenced. The proportions of high-quality sequences in FTLS and control pools were 98.08% (10,198,407/10,397,161) and 97.88% (10,250,809/10,472,315), respectively. Unique, high-quality sequence reads were then classified into broad taxonomic groups based on the taxonomy of the most frequent top-scoring BLAST matches for each sequence. Virus sequences constituted 0.0065% (67,969) and 0.0048% (50,677) of the reads in FTLS and control libraries, respectively (Table 3). After contig assembly, the number of sequences phylotyped as “viral” decreased to 3,163 and 2,412, respectively, in the two pools (Table 4). To screen for possible viruses, we focused exclusively on viruses that were present in patient sera.

**Table 3:** Categorization of sequence reads based on SOAP criteria.

sample	raw reads data	valid reads data	Reads removed host	viruses reads data
Patients' Sera	10397161	10198407	3709639	67969
Sera control	10472315	10250809	3449274	50677

Criteria for SOAP including ID≥80%, E-value≤1e-2 and Match query≥0.7.

For detection of known viruses, non-redundant reads were directly aligned with the GenBank database of nucleic acids using BLASTn software. Some sequences from *Adenoviridae* (human adenovirus), *Herpesviridae* (herpesvirus), *Papillomaviridae* (papillomavirus) and *Retroviridae* (human endogenous retrovirus) were detected in both FTLS patients and healthy subject samples. Some hepatitis B virus (HBV) and human bocavirus sequences were detected only in patient sera, indicating the presence of HBV and human bocavirus infections among these patients. To detect novel viruses, we examined sequence data against the GenBank protein database using BLASTx. An analysis of the deduced protein sequences revealed four different virus families in sera from FTLS patients (Table 4). Among these were viruses from *Hepadnaviridae*, which are not known to cause FTLS; Torque teno virus (TTV) from *Anelloviridae*, which has been reported to be associated with certain inflammatory states [34], but is not known to be transmitted by arthropods; and viruses from the *Parvoviridae* family, including human bocavirus, which could cause febrile illness and signs of FTLS. Although human bocaviruses are not known to be transmitted by arthropods, feline panleukopenia virus, a parvovirus, is strongly suspected to be transmitted by arthropods [35]. All family *Parvoviridae* sequences detected in FTLS samples were also assembled and their protein sequences deduced. Included among these samples were four fragments, all of which were found to be highly homologous to human bocavirus; one fragment showed the greatest similarity to human bocavirus 2 isolate 53044 (identity  = 86%) with the lowest E-value (8×10^−17^). Human bocavirus was further detected by PCR in the pool of 10 serum samples, but only one individual sample within the pool tested positive for bocavirus. The final virus family detected in sera from FTLS patients was the *Bunyaviridae* family, which contains viruses known to cause FTLS after tick bites [8], [30]. All family *Bunyaviridae* sequences detected in FTLS samples, including 11 fragments (1 S-segment, 2 M-segments, and 8 L-segment fragments), were assembled and their protein sequences were deduced. Among these 11 novel virus fragments was a 168-bp fragment (C361, Accession: HQ412604) of the polymerase gene that showed the greatest similarity to Tehran virus (identity  = 36%) with the lowest E-value (3×10^−7^). This suggested the presence of a novel virus or a known virus whose genome had not yet been sequenced.

**Table 4:** sequence reads with limited BLASTx identity to known viruses.

Virus family*	Genus/Species Name (Top Hit)	Sera from patients	Sera from healthy people
		Read Counts	Read Counts
**Anelloviridae**	Alphatorquevirus	185	
	Gammatorquevirus	11	
	Iotatorquevirus	16	
	Thetatorquevirus	2	
**Bunyaviridae**	Hantaan virus	11	
	Nairovirus	160	
	Orthobunyavirus	43	
	Phlebovirus	121	
	Tospovirus	5	
**Hepadnaviridae**	Hepatitis B virus	230	
**Parvoviridae**	Human bocavirus	12	
	Aleutian mink disease virus	2	
**Astroviridae**	Human astrovirus		22
**Adenoviridae**	Atadenovirus	18	
	Aviadenovirus	24	13
	Ichtadenovirus		11
	Mastadenovirus	142	529
	Siadenovirus	7	85
**Alloherpesviridae**	Cyprinid herpesvirus 1	85	100
**Arenaviridae**	Lymphocytic choriomeningitis virus	47	22
**Herpesviridae**	Cytomegalovirus	18	
	Lymphocryptovirus	31	
	Iltovirus		49
	Macavirus		16
	Muromegalovirus	28	3
	Percavirus	35	21
	Rhadinovirus	81	
	Simplexvirus	93	141
	Varicellovirus	273	211
**Papillomaviridae**	Human papillomavirus 10	24	137
**Paramyxoviridae**	Human metapneumovirus	14	4
	Human respiratory syncytial virus	2	83
**Retroviridae**	Alpharetrovirus	651	273
	Betaretrovirus	337	
	Deltaretrovirus	289	87
	Gammaretrovirus	166	568
	Epsilonretrovirus		37

*Bold indicates virus families only found in FTLS patient sera. An E-value cutoff of 1e-5 for BLASTx was applied.

**Figure 1:**
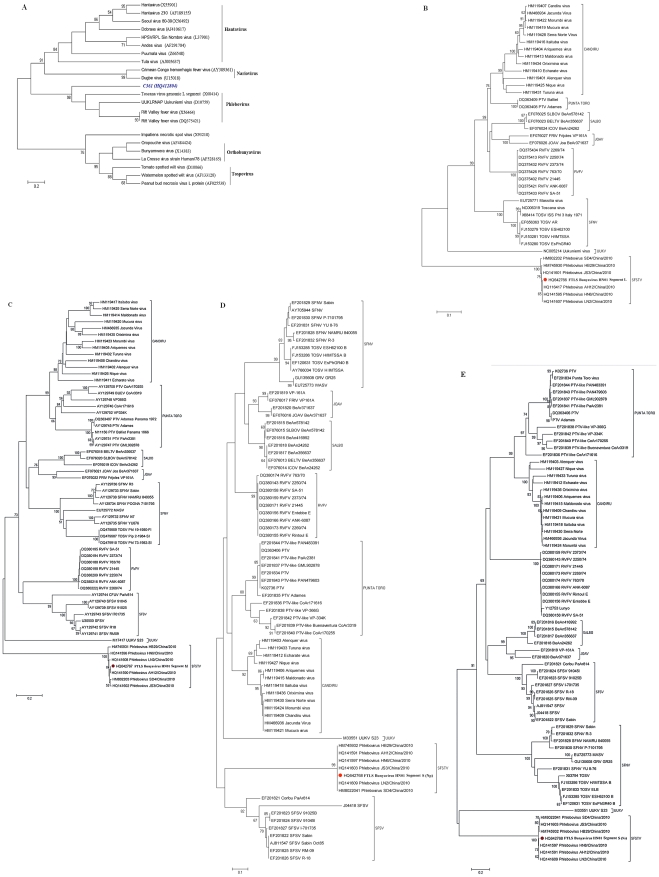
Phylogenetic analysis of novel bunyavirus proteins. Phylogenetic trees were generated by comparing the translated amino acid sequences of individual sequence reads to the corresponding sequences from known bunyaviruses using a neighbor-joining method. The horizontal-line distance represents the number of sites at which the two compared sequences are different. Bootstrap values deduced from 1000 replicates. A: C361 fragment (Accession number HQ412604). B: FTLS bunyavirus (Henan isolate), Segment L (Accession number HQ642766). C: FTLS bunyavirus (Henan isolate), Segment M (Accession number HQ642767). D: FTLS bunyavirus (Henan isolate), Segment S (Np) (Accession number HQ642768). E: FTLS bunyavirus (Henan isolate), Segment S (Ns) (Accession number HQ642768).

To more accurately assess the genetic relationships to known viruses, we constructed a phylogenetic tree using a neighbor-joining method [33]. The result showed that the potentially novel virus clustered with Toscana virus, Uukuniemi virus, and Rift Valley fever virus of the *Phlebovirus* genus (Figure 1A). The pools of 10 serum samples from patients and 10 serum samples from healthy subjects were also screened by PCR for the presence of the novel bunyavirus. All 10 samples from patients tested positive for the novel bunyavirus; however, all 10 samples from healthy subjects tested negative. Thus, we further focused on the virus from the family *Bunyaviridae*.

### Confirmation of the presence of the novel virus in patients' sera

Using specifically designed RT-PCR primers, we detected viral RNA in 223 of the 285 acute serum samples tested (Table 5). The specificity of the RT-PCR was confirmed by sequencing selected PCR products. None of the 80 sera from patients with respiratory diseases or the 50 sera from healthy subjects was positive using the novel virus-specific RT-PCR.

**Table 5:** Detection of novel FTLS bunyavirus-specific IgG antibody and viral RNA by IFA and RT-PCR.

Course of Disease^a^	Number of Samples	Number of Positive	
		IgG (%)	RNA (%)
1–3	87	4 (4.59)	72 (82.75)
4–6	134	27 (20.14)	117 (87.31)
7–14	64	49 (76.56)	34 (53.12)
Total	285	80 (28.07)	223 (78.24)

a : Days were counted based on the onset of symptoms.

### Isolation of the novel virus

**Figure 2:**
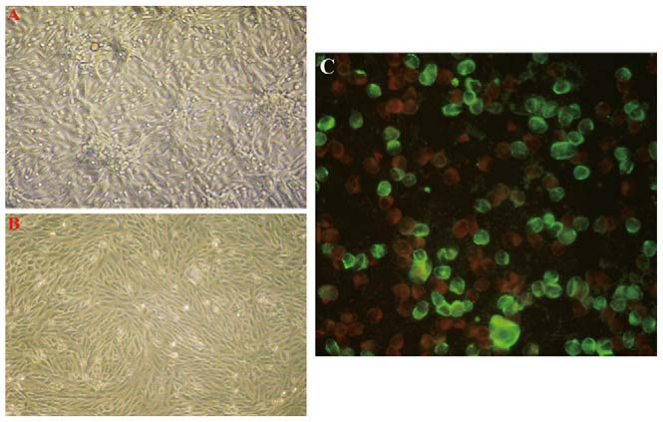
Vero E6 cells inoculated with acute serum specimens from patients with FTLS. The typical early CPE seen with novel bunyavirus isolates from patients with FTLS is shown in part A (×40). Mock-inoculated Vero cells are shown in part B (×40). Infected Vero cells reacted with the serum of a convalescent FTLS patient in an indirect IFA in part C (×400).

**Figure 3:**
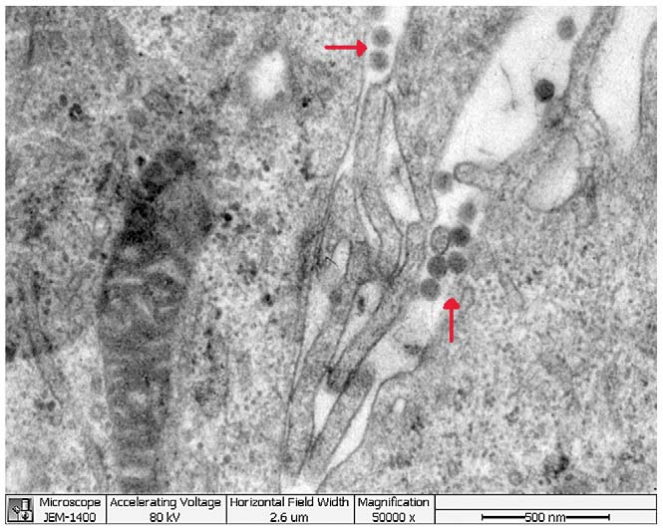
Thin-section electron microscopy of novel FTLS-associated bunyavirus Grown in Vero E6 Cells.

Six acute serum samples that tested positive for the novel bunyavirus by specific RT-PCR were inoculated onto Vero E6 cells, and four virus strains were isolated. The initial CPE analysis showed rounded refractile cells 2–4 days after inoculation. CPE did not progress in the initial cultures, but appeared slightly at 24 hours in subsequent passages (Figure 2A). RT-PCR revealed the presence of RNA for the novel bunyavirus in all four virus strains, and all isolates reacted with the serum of a convalescent patient in IFA (Figure 2C). In addition, electron microscopy showed the presence of virus particles approximately 80–90 nm in diameter—a size compatible with a bunyavirus (Figure 3). Virus particles were presumably localized to the Golgi apparatus (Figure 3).

### Confirmation of the isolated virus by direct sequencing

Genome sequencing of one isolate (HN01) revealed three segments of negative polarity, single-stranded RNA, including a large segment (L; GenBank HQ642766), a medium-sized segment (M; GenBank HQ642767), and a small segment (S; GenBank HQ642768). The deduced amino acid sequence of the L segment had the highest homology (34%, E value  = 3×10^−163^) to RNA polymerase of the Uukuniemi virus of the *Phlebovirus* genus, whereas the M segment had the highest homology (26%, E value  = 8×10^−55^) to glycoprotein genes of the Punta Toro virus in the *Phlebovirus* genus. Of the two proteins encoded by the ambisense S segment, one had the highest homology to nucleocapsid protein (39%, E value  = 3×10^−40^) of the Rift Valley Fever virus, and the other had the highest homology to nonstructural protein genes (24%, E value  = 0.049) of the Punique Virus, both of which belong to the *Phlebovirus* genus. Collectively, these findings confirm that this virus belongs to the *Phlebovirus* genus of *Bunyaviridae*.

During the revision of this manuscript, some new sequences of SFTSV (severe fever with thrombocytopenia syndrome) were released. A comparison of these new SFTSV sequences with the sequence of this novel virus showed that they were highly homologous (>99% identity). Using sequences of *Phlebovirus* available in GenBank, a phylogenetic analysis showed that, although most closely related to the Uukuniemi virus of the *Phlebovirus* genus (34%, 24%, and 29% of maximum identity, respectively, for segment L, M, S at maximum query coverage), the three genomic segments of the novel virus, along with the SFTSV sequences, were highly divergent (Figure 1B–1E).

### Antibody response to the bunyavirus

IgG antibodies to the novel bunyavirus were detected in 80 of 285 acute-phase serum samples from patients with FTLS (Table 5). Of 95 patients from whom paired acute- and convalescent-phase sera were available, 52 had seroconversions and 21 had greater than 4-fold increases in antibody titer to the virus. Six had less than a 4-fold increase in antibody titer to the virus, but all paired sera tested positive. Sixteen patients tested negative to the virus, suggesting that some non-FTLS patients with similar symptoms were included in this study, a situation that is not surprising given that FTLS is a newly emerging disease. The acute-phase sera of four patients from whom the virus was isolated tested negative for IgG antibody to the virus. All convalescent sera obtained 2 months later from the same four patients contained IgG antibody to the virus. None of the 130 sera from patients with respiratory diseases or healthy subjects had detectable antibody.

## Discussion

Since 2007, there has been an increase in reported cases of FTLS in Xinyang City, Henan Province. These patients were tentatively diagnosed as having *A. phagocytophilum* infection. However, only a few (8.4%, 24/285) such patients had evidence for *A. phagocytophilum* infection, and none of the 285 patients tested positive for the many other pathogens capable of causing similar clinical and laboratory manifestations that were also investigated. These findings suggested novel infectious agents, including viruses.

Traditionally, virus culture is very important for identifying an unknown viral infection. Before performing the Illumina sequencing strategy, we attempted viral and rickettsial culture with DH82 and BHK cell lines, but the lack of an obvious CPE led us to initially abandon this approach. Here, mass sequence data obtained by Illumina sequencing revealed four virus families that appeared only in FTLS patient sera. Among these four virus families, viruses from the *Parvoviridae* and *Bunyaviridae* families reportedly can cause signs of FTLS and be transmitted by arthropods. However, only one sample from a pool of ten samples tested positive for bocavirus by PCR, suggesting that bocavirus from the *Parvoviridae* is not likely involved in FTLS. For viruses in the *Bunyaviridae* family, the incidence of infection is closely linked to vector activity. For example, tick-borne viruses are more common in the late spring and late summer when tick activity peaks. Human infections with certain *Bunyaviridae*, such as Crimean-Congo hemorrhagic fever virus, are associated with high levels of morbidity and mortality [30]. Considering the tick-bite history of many FTLS patients, we focused on *Bunyaviridae* family viruses.

The entire *Bunyaviridae* family contains more than 300 members arranged in four genera of arthropod-borne viruses (*Orthobunyavirus*, *Nairovirus*, *Phlebovirus* and *Tospovirus*) and one genus (*Hantavirus*) of rodent-borne viruses [30], [36]. The *Phlebovirus* genus currently comprises 68 antigenically distinct serotypes, only a few of which have been studied. The 68 known serotypes are divided into two groups: the Phlebotomus fever group (the sandfly group, transmitted by *Phlebotominae* sandflies) comprises 55 members, and the Uukuniemi group (transmitted by ticks) comprises the remaining 13 members. Of these 68 serotypes, eight are linked to disease in humans, including the Alenquer, Candiru, Chagres, Naples, Punta Toro, Rift Valley fever, Sicilian, and Toscana viruses [30]. Phleboviruses have tripartite genomes consisting of a large (L), medium (M), and small (S) RNA segment.

In screening for unknown viruses, species hits alone likely carry little weight. Thus, we used all sequences in the family *Bunyaviridae* for our analysis. A 168-bp fragment of the polymerase gene with the lowest E-value and high sequence identity was used as the sequence of the unknown virus. This virus sequence was detected in all 10 pooled samples, indicating that the virus is involved in FTLS. After detecting a possible novel bunyavirus through high-throughput Illumina sequencing, we inoculated Vero cell lines, which are known to be sensitive to phleboviruses, with sera from six positive patients and were subsequently able to detect the virus by RT-PCR [30], [31]. Although the CPE was modest, RT-PCR confirmed the infection. Genome sequencing was performed and a phylogenetic analysis of the genome sequence showed that this virus clustered into the *Phlebovirus* branch, but was divergent from other known phleboviruses. These results confirm the novelty of this virus within the *Phlebovirus* genus of the family *Bunyaviridae* [36]. Furthermore, virus size and propagation in cells were similar to that of the bunyaviruses.

PCR and serological tests were performed to further test the causal link between the new virus and FTLS. Although we have not completely fulfilled Koch's postulates, evidence implicating this new bunyavirus in the outbreak of the disease among patients with FTLS is compelling.

In view of the fact that the disease is caused by a novel bunyavirus, and taking into account that the disease was first discovered in Henan (HN), we propose the name "Henan Fever" for the FTLS disease cause by the novel virus (proposed name “Henan Fever Virus” [HNF virus]). Since the submission of this manuscript, a bunyavirus was identified as the cause of FTLS in Chinese patients from other regions of China, and the authors have named this virus “SFTSV” to indicate that it is the cause of severe fever with thrombocytopenia syndrome [37]. After release of the GenBank sequences referred to in the Yu paper, we compared the sequences of SFTSV with those of FTLSV and found that they were nearly identical (>99% identity). As we first identified the syndrome in 2007 and described the presence of the virus in patients between 2007 and 2010, we suggest that the name “HNF virus” should take precedence. The most distinctive feature of the current work includes the use of an unbiased metagenomic approach for viral pathogen discovery that facilitated the rapid creation and implementation of standard culture, serological, and molecular diagnostic approaches. However, there are other differences between the results described here and those reported by Yu et al; notably, we observed slight, but distinctive, CPE in Vero cells. The reason for the failure to observe CPE in Vero cells infected with the “SFTSV” bunyavirus [37], whose genome is nearly identical to that of bunyavirus isolated from our FTLS patients, is unclear. Perhaps this reflects the fact that the ensuing CPE is not dramatic. Alternately, this could indicate the existence of distinct viral strains that vary in pathogenicity, virulence, and possibly even disease manifestations. This is an area of active study in our laboratories.

The discovery of this new virus will assist in the rapid diagnosis of this disease and help to distinguish it from other diseases caused by pathogens such as *A. phagocytophilum*, *E. chaffeensis*, Crimean-Congo hemorrhagic fever virus, Hantavirus, dengue virus, Japanese encephalitis virus, and Chikungunya virus. Furthermore, the availability of the new virus will facilitate the future development of new therapeutic interventions, such as vaccines and drugs.

## Supporting Information

Figure S1 Sensitivity and dynamic range of real-time PCR in the detection of bunyavirus RNA. To evaluate sensitivity of our RT-PCR, a real-time PCR was performed. Serial dilutions of in vitrobtranscribed bunyavirus RNA sequences were tested. A wide linear range (from 5 copies to 5×10^7^ copies of control RNA per reaction) was detected in this assay.
(TIF)
