# Supplementary figures and images for: Metagenomic Analysis of Fever, Thrombocytopenia and Leukopenia Syndrome (FTLS) in Henan Province, China: Discovery of a New Bunyavirus
Source: PLoS Pathog. 2011 Nov 17;7(11):e1002369. doi: 10.1371/journal.ppat.1002369 (PMC3219706; doi:10.1371/journal.ppat.1002369)

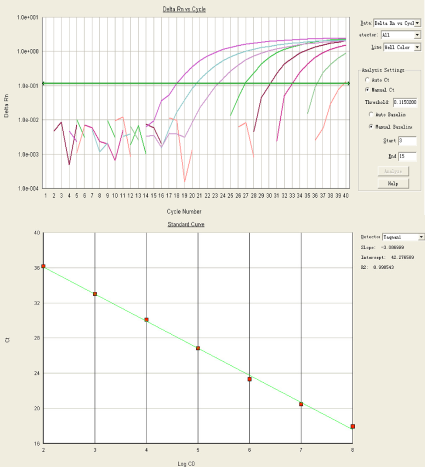

Supplement: Figure S1 — Sensitivity and dynamic range of real-time PCR in the detection of bunyavirus RNA. To evaluate sensitivity of our RT-PCR, a real-time PCR was performed. Serial dilutions of in vitrobtranscribed bunyavirus RNA sequences were tested. A wide linear range (from 5 copies to 5×10^7^ copies of control RNA per reaction) was detected in this assay. (TIF) [file ppat.1002369.s001.tif]
